# A study of clinical, hematological, and biochemical profiles of patients with dengue viral infections in Northwest Ethiopia: implications for patient management

**DOI:** 10.1186/s12879-018-3557-z

**Published:** 2018-12-04

**Authors:** Getachew Ferede, Moges Tiruneh, Ebba Abate, Yitayih Wondimeneh, Endalamaw Gadisa, Rawleigh Howe, Abraham Aseffa, Belay Tessema

**Affiliations:** 1 Department of Medical Microbiology, School of Biomedical and Laboratory Sciences, College of Medicine and Health Sciences, University of Gondar, Gondar, Ethiopia; 2 Ethiopian Public Health Institute, Addis Ababa, Ethiopia; 3 Armauer Hansen Research Institute, Addis Ababa, Ethiopia

**Keywords:** Dengue, Clinical features, Hematological tests, Biochemical parameters

## Abstract

**Background:**

Dengue is one of the most common arboviral diseases with increased outbreaks annually in tropical and subtropical areas. In Ethiopia, there are no data regarding clinical, hematological and biochemical parameters which are very important in the clinical management of dengue patients. Hence this study was carried out to provide the first baseline data of clinical, hematological and biochemical profiles of patients infected with dengue virus.

**Methods:**

A cross-sectional study was carried out among febrile patients in northwest Ethiopia from March 2016 to May 2017. Blood samples were collected from dengue presumed cases and tested against dengue specific IgM antibody by enzyme-linked immunosorbent assay (ELISA). Those study participants who fulfilled the inclusion criteria were enrolled in the study. Clinical examination findings were recorded, hematological and biochemical parameters tests were done.

**Results:**

During the study period, a total of 102 dengue cases were included in the study. Of these, there were 16 (15.7%) children and 86 (84.3%) adults between 1 and 76 year age. The most common clinical presentations followed by fever (100%) were a headache 89 (87.3%), myalgia 82 (80.4%), nausea/vomiting 71 (69.6%). The common hematological findings were thrombocytopenia 61 (59.8%), followed by anemia 45 (44.1%) and leucopenia 27 (26.5%) and the elevated levels of biochemical parameters were AST 46 (45.1%) and ALT in 18 (17.6%).

**Conclusions:**

This study highlights the most common clinical and laboratory profiles of dengue viral infections that could alert physicians to the likelihood of dengue virus infections in the study area.

## Background

Dengue is a mosquito-borne arboviral disease and is a major global public health threat that is prevalent in tropical and sub-tropical regions of the world, mostly in urban and semi-urban areas [1]. The WHO estimates, more than 2.5 billion individuals live at risk of dengue transmission in more than 100 countries and approximately 50-100 million individuals have infected with dengue annually [2]. Of these, 500,000 severe dengue cases are diagnosed each year resulting in 24,000 deaths per year [3–6]. Dengue is endemic in the WHO regions of Africa, the Americas, the Eastern Mediterranean, South-East Asia, and the Western Pacific regions and the Caribbean [7]. In the recent decades, the global incidence of dengue virus (DENV) infection has increased with increasing geographic expansion to new countries [8]. The threat of a possible outbreak of dengue for the first time reported from Ethiopia in 2013 in Dire Dawa. In this outbreak, 11, 409 dengue suspected cases within four months were reported. Fifty (50/88; 56.8%) samples were reported positive for dengue infection and mostly affected age groups were 15-44 year-olds [9]. Dengue cases were also later reported in 2014 from Somali Regional state of Ethiopia, in which 33 (33/57, 57.9%) samples were reported positive for dengue [10].

The leading contributing factors for widespread and increasing dengue incidences are rapid unplanned urbanization and migration of population to urban areas, poor sanitation facilities contributing fertile breeding areas for mosquitoes, lack of vector control and climatic changes [11]. As mosquitoes are widely distributed in Africa and can serve as vectors of dengue virus, when these combined with rapid population growth, unplanned urbanization, and increased international travel, could increase the epidemic risk in African countries [12].

Specific treatment for dengue is not available, but early detection and fluid replacement therapy and use of analgesics and antipyretics with good nursing care ensures marked reduction of the mortality rates 20% to less than 1% due to severe cases [13]. In clinical practice, as it is known, the patient's diagnosis and management are based on clinical manifestations and abnormal laboratory findings [14]. Initial DENV infection may be asymptomatic [15] or may result in a nonspecific febrile illness typically present with the sudden onset of fever, severe headache, bone, joint and muscular pains, mild bleeding manifestation, weakness, myalgia, and rash [16]. All these clinical presentations are similar to many other febrile diseases prevalent in the country; such as malaria, Kala-azar and typhoid fever which pose a diagnostic challenge of dengue [17].

Dengue is caused by infection with any one of the four serotypes of DENV which is an arbovirus single-stranded RNA virus of the genus Flavivirus [18]. Infection with one dengue serotype provides lifelong homotypic immunity to that particular serotype. Different serotypes can be in circulation during an epidemic, and thus, a person can eventually be infected as many as four times, once with each serotype [19]. It is well documented that subsequent infection with different DENV serotypes increases the risk of developing severe dengue [20, 21]. Infection with dengue can be diagnosed by using clinical presentations and laboratory tests. Of the laboratory tests, non-specific tests; like hematological parameters, liver function tests, and serum protein concentration and specific tests; such as viral antigen test, genomic sequence, and serology for antibody detection are used [22, 23].

Dengue was noticed recently in Ethiopia but there is no data concerning clinical and laboratory profiles of the cases with dengue in the country. Physicians should be aware of the most common clinical as well as hematological and biochemical presentations which are important for the clinical management of patients and thus crucial for saving a life. Therefore, this study aimed to highlight the most common clinical features, hematological and biochemical findings of dengue cases.

## Methods

### Study area and participants

A cross-sectional prospective, hospital-based study was carried out in Metema and Humera Kahsay Abera hospitals, northwest Ethiopia from March 2016 to May 2017.

### Inclusion criteria

Febrile patients who were presumed for dengue infection based on 2009 WHO criteria [8] and serologically confirmed with dengue specific IgM antibody. The febrile patient is referred to one whose axillary temperature is ≥ 38 ^o^C.

### Exclusion criteria

Cases confirmed as malaria, Kala-azar, typhoid fever, and any other confirmed chronic diseases were excluded in the study.

### Data collection

Clinical examinations were performed by a physician on each study participant. Demographic variables, as it has been published in previous work, [24] and clinical profiles of study participants were collected by nurses using the structured questionnaire. The diagnosis of dengue was made based on positive enzyme-linked immunosorbent (ELISA; manufactured by EUROIMMUN diagnostics) [25] assay result for specific IgM antibody for dengue in serum. All the routine investigations such as hematological determination like total leukocyte count (TLC), differential leukocyte count, platelet count; hemoglobin (Hgb) and hematocrit (Hct) were determined by the automated blood analyzer (CELL-DYN 1800, Abbott Laboratories Diagnostics Division, USA). Thick and thin blood smear for malaria parasite, biochemical tests; AST and ALT for liver function tests, creatinine, and BUN for renal function tests and total protein were done by the automated biochemistry analyzer (Vegasys) [26]. The cutoff values of each test results were considered based on reference ranges used by the laboratory. Furthermore, medical charts of all dengue specific IgM positive cases were reviewed for the collection of other information (i.e. Kala-azar, typhoid fever, and any other confirmed chronic cases).

### Statistical analysis

Data were entered and analyzed using the SPSS 20.0 statistical software. Descriptive statistic was used to calculate frequency and percentage. Data were presented by using tables and figure.

## Results

### Clinical profiles of dengue cases during a hospital visit

**Fig. 1:**
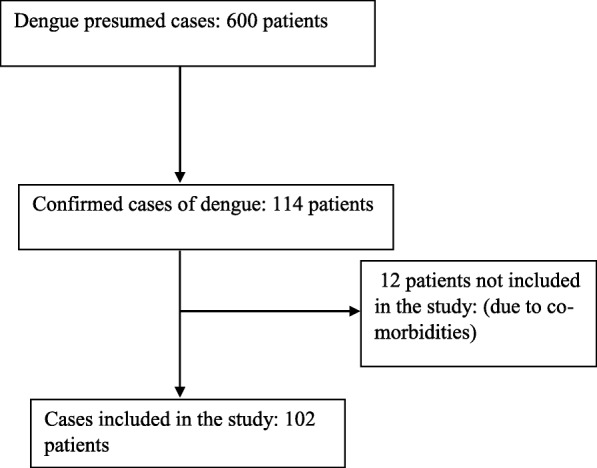
Flow chart of the study

During the study period, 600 febrile patients who were presumed for dengue viral infections based on 2009 the WHO criteria were tested for dengue-specific IgM antibody. Of these, 114 patients were positive for dengue. Out of positive cases, 102 (89.5%) were included in this study while the remaining 12 (10.5%) were not included in the study due to co-morbidities (Fig. 1). Seventy-eight (78/102; 76.5%) study participants were males. A study participants’ age varied from 1 year to 76 years. Eighty sex (86/102; 84.3%) study participants were ≥ 15 years (Table 1). The commonest clinical feature was fever, 102 (100%), followed by headache in 89 (89/102; 87.3%), myalgia 82 (82/102; 80.4%), nausea/vomiting 71 (71/102; 69.6%), abdominal pain 61 (61/102; 59.8%), eye pain and mucosal bleeding 38 (38/102; 37.3%) in each of the cases, conjunctival hemorrhage and hepatomegaly 23 (23/102, 22.5%) in each of the cases. Rashes and tourniquet test in 13 (13/102; 12.7%) and 8 (8/102; 7.8%) of cases were seen, respectively (Table 2).

**Table 1:** Gender and age of the study participants (*N* = 102)

Variables	Number of participants	Percentage
Gender		
Male	78	76.5
Female	24	23.5
Age		
< 15 years	16	15.7
≥ 15 years	86	84.3

**Table 2:** Clinical presentations of dengue cases at the time of hospital visit (*N* = 102)

Clinical feature	Number of cases	Percentage
Fever	102	100
Abdominal distention	14	13.7
Headache	89	87.3
Eye pain	38	37.3
Myalgia	82	80.4
Mucosal bleeding	38	37.3
Abdominal pain	61	59.8
Nausea and vomiting	71	69.6
Rash	13	12.7
Tourniquet test	8	7.8
Conjunctival hemorrhage	23	22.5
Hepatomegaly	23	22.5

### Hematological profiles of dengue cases during a hospital visit

The most common hematological finding observed was thrombocytopenia (platelet count < 140,000/cumm) in 61 (61/102; 59.8%), followed by anemia (hemoglobin level < 11.5 g/dl) in 45 (45/102; 44.1%) and leucopenia (total leukocyte count < 4,000/cumm) in 27 (27/102; 26.5%) of the cases. Hematocrit > 44% were noted in 10 (10/102; 9.8%) of the cases and it ranged between 9-51%. Neutrophil < 1500 in 16 (16/102; 15.7%) and lymphocyte > 2900 in 2 (2/102; 2%) of the cases were noticed (Table 3).

**Table 3:** Hematological parameters of dengue cases at the time of hospital visit (*N* = 102)

Hematological test	No. of patient N (%)	Normal lab value
Platelet count (cells/cumm)		140,000-415,000/cumm
< 140,000	61 (59.8)	
≥140,000	41 (40.2)	
WBC count (cells/cumm)		4,000-10,500/cumm
< 4,000	27 (26.5)	
≥ 4,000	75 (73.5)	
Hemoglobin g/dl		
Male ≤ 13	34 (43.6)	M: 13-16
Female ≤ 12	11 (45.8)	F: 12-15
Sub-total	45 (44.1)	
Hematocrit (%)		
Male > 46	8 (10.3)	M: 38-46
Female > 44	2 (8.3)	F: 35-44
Sub-total	10 (9.8)	
Neutrophil (cells/cumm)		1500-8000
< 1500	16 (15.7)	
≥ 1500	86 (84.3)	
Lymphocyte (cells/cumm)		900-2900
≤ 2900	100 (98)	
> 2900	2 (2)	

### Biochemical parameters of dengue cases at the time of hospital visit

The results of the biochemical investigation revealed that alanine aminotransferase (ALT) level > 42 IU/L was observed in 18 (18/102; 17.6%) of cases and aspartate aminotransferase (AST) level > 37 IU/L observed in 46 (46/102; 45.1%) of cases. The levels of ALT and AST ranged between 4-347 IU/L and 8-320 IU/L, respectively. Creatinine level > 1.1 mg/dl was observed in 20 (20/102; 19.6%) and BUN > 23.5 mg/dl noted in 15 (15/102; 14.7%) of cases. Hypoproteinaemia (total protein value < 6.6 mg/dl) was observed in 22 (22/102; 21.6%) cases (Table 4).

**Table 4:** Biochemical parameters among dengue cases (*N* = 102)

Parameter	No. of cases N (%)	Normal lab value
ALT (IU/L)		3-42 IU/L
≤ 42	84 (82.4)	
> 42	18 (17.6)	
AST (IU/L)		5-37 IU/L
≤ 37	56 (54.9)	
> 37	46 (45.1)	
Creatinine (mg/dl)		0.6-1.1 mg/dl
≤ 1.1	82 (80.4)	
> 1.1	20 (19.6)	
BUN (mg/dl)		4.7-23.5 mg/dl
≤ 23.5	87 (85.3)	
> 23.5	15 (14.7)	
Total protein (mg/dl)		6.6-8.7mg/dl
< 6.6	22 (21.6)	
≥ 6.6	80 (78.4)	

## Discussion

In recent decades, the global prevalence of dengue has increased dramatically due to the limitations of currently available control strategies, such as vaccines and pesticides [27, 28]. Hence early diagnosis and proper medical management are of prime importance. As dengue is a recently known problem in Ethiopia [9, 10], the knowledge regarding its clinical presentations along with laboratory tests is vital for patient management. Hence, this study emphasized to document the first baseline data on clinical manifestations, hematological and biochemical parameters of patients with dengue illness in the country. The evidence generated is crucial for proper management of dengue patients.

In this study, the most frequent clinical presentation was fever, followed by a headache, myalgia, nausea/vomiting and abdominal pain, which is in agreement with the other studies [29, 30]. Rash was seen in 12.7% and hepatomegaly in 22.5% of the cases in our study while in another similar study 28.1% and 12.5% of patients presented with rash and hepatomegaly, respectively [31]. Bleeding diathesis is a common clinical presentation of dengue due to low platelet count and leakage from blood vessels. This is due to the interaction of dengue virus with host cells which cause the release of excess cytokines and stimulation of immunologic mechanism causing vascular endothelial alteration, infiltration of mononuclear cells and perivascular edema [32]. In our study mucosal bleeding was observed in most of the patients, which is in agreement with the other study [33], but in contrast to another study which showed cutaneous bleeding as the most common hemorrhagic manifestation [34]. In another study, hemorrhagic manifestation in the form of petechiae only was reported [31]. The variations in the clinical presentation of different studies might be due to the difference in the strain of the virus and its virulence factor.

Among the hematological profiles, thrombocytopenia was the most common finding in the present study which is consistent with the other studies [35, 36]. Thrombocytopenia might be due to decreased production of platelets due to suppression of the bone marrow by a virus and also due to binding of dengue antigens to platelets and increased antibody mediated immunological destruction of platelets [37, 38]. Leucopenia is one of the hematological parameters which occurs in people who were afflicted with dengue bone marrow suppression [38]. In our study, leucopenia was observed in 26.5% of the cases while in other studies elsewhere leucopenia was observed in 56.9% [39] and in 50% of the cases [40].

Hemoglobin levels less than the cutoff values were observed in 45 (44.1%) of the cases. This could be due to mucosal bleeding in which 38 (37.3%) of the cases had bleeding. This finding is consistent with another study [41]. Increased hematocrit was observed in 6.9% of cases in our study while it is less than the observation of the others studies which was reported 50% [42] and 27% [40] rise in hematocrit value. This might be related to increased severity and is elucidated by the hemoconcentration due to increased intravascular plasma permeability which is the basic pathophysiological changes in dengue.

In this study, higher levels than the normal values of AST in 45.1% and ALT in 17.6% of the cases were observed which showed elevated levels of AST in a greater proportion of cases than ALT. This is in agreement with the other studies elsewhere that reported AST and ALT higher levels in 68.5% and 39.2% of the cases, respectively [43] and similar findings were also reported elevated AST in 72.7% and ALT in 27.3% of cases elsewhere [44]. Dengue virus is hepatotrophic and also damages other organs; hence the observed pattern could be explained due to excess release of AST from damaged muscle cells (nonhepatic source) during infection that leads to more deranged AST than ALT. The ALT is mainly associated with hepatocytes, with the smallest activity in cardiac and skeletal muscle and AST is found in erythrocytes, kidney and brain tissue, cardiac and skeletal muscle and is usually raised because of damage to those sources and response to hepatic damage [45].

The serum creatinine and blood urea (BUN) levels were raised in 19.6% and 14.7% of the cases, respectively. These could be due to a direct viral effect on the glomerular and tubular cells or as a result of tissue injury caused by deregulated host immune response against the viral antigens [46]. Hypoproteinemia was observed in 21.6% of the cases. This could be probable that the complex interaction between virus, host immune response and endothelial cells, may affect the barrier integrity and functions of the vascular endothelial cells leading to plasma leakage causing hypoproteinemia [47].

Although this is the first effort to study clinical, hematological and biochemical profiles of dengue viral infection in Ethiopia, the study has several limitations. Comparisons of those dengue cases with the control group and serial hematological and biochemical changes were not done due to the nature of cross-sectional study design. These could be done in the future for better understanding of dengue viral infection presentation. Regardless of the depicted limitations, this preliminary study ultimately provides the first baseline data on clinical and laboratory profiles of dengue viral infection in the country.

## Conclusion

Awareness of clinical features, as well as laboratory findings like hematological and biochemical parameters, are the most important guide to therapy and prognosis of dengue. In this study, fever followed by a headache and myalgia were commonest presenting complaints of dengue patients. Thrombocytopenia, anemia, leucopenia and elevated levels of AST were identified as the most common findings. Thus, these findings should prompt physicians on the probability of dengue infection in the study area.

## Abbreviations

ALT: Alanine aminotransferase
AST: Aspartate aminotransferase
BUN: Blood Urea
DENV: Dengue Virus
ELISA: Enzyme-Linked Immunosorbent Assay
HCT: Hematocrit
IgM: Immunoglobulin M
